# Construction of an Integrated mCherry Red Fluorescent Protein Expression System for Labeling and Tracing in *Lactiplantibacillus plantarum* WCFS1

**DOI:** 10.3389/fmicb.2021.690270

**Published:** 2021-06-22

**Authors:** Yao Yang, Wenjun Zhang, Hailin Huan, Wenxu Xia, Ying Chen, Peijuan Wang, Yanrong Liu

**Affiliations:** ^1^School of Food Science and Pharmaceutical Engineering, Nanjing Normal University, Nanjing, China; ^2^Jiangsu Academy of Agricultural Sciences, Nanjing, China; ^3^Geneception (Shanghai) Bio-technology Co., Ltd., Shanghai, China; ^4^Nanjing Institute of Product Quality Inspection, Nanjing, China

**Keywords:** mCherry fluorescent protein, probiotic tracking, probiotic labeling, *Lactiplantibacillus plantarum* WCFS1, fluorescent reporter system

## Abstract

Thorough intestinal adhesion and colonization greatly promote the probiotic properties of lactic acid bacteria (LAB). Labeling and tracing with fluorescent proteins are effective and reliable for studying the *in vivo* physiological activities of LAB including localization, adhesion, and colonization. *Lactiplantibacillus plantarum* WCFS1 was successfully traced with a red fluorescent protein (RFP), which was expressed by the bacteria-carrying recombinant plasmids. In this study, we aimed to construct a stable RFP mCherry expression system, whose encoding gene was integrated into the bacterial chromosome *via* double-crossed homologous recombination, and use it for labeling WCFS1 with the goal of avoiding the potential loss of non-chromosomal plasmids along with intestinal growth. First, the constitutive expression of the mCherry protein was improved after adjusting the length of the spacer between the promoter and the gene start codon. Then, the optimized mCherry gene expression cassette was integrated into the chromosome of WCFS1. The resulting strain had normal unimpaired growth and strong fluorescent signals, even after 100 generations, indicating its stability. Furthermore, quantitative polymerase chain reaction (PCR) results revealed a strong positive correlation between the fluorescence intensity of the strain and the number of viable cells, demonstrating its potential usage for the quantification of *in vivo* WCFS1 cells. Finally, the increased adhesion ability of WCFS1 due to the recombinant expression of the *bsh* gene was visualized and evaluated using fluorescence intensity, the results of which were consistent with those obtained using the previously established quantification methods. These results suggest that the chromosomal-integrated mCherry labeling system can be extensively used to examine the distribution, colonization, and survival of LAB *in vivo* in order to determine the mechanism of its probiotic function.

## Introduction

Lactic acid bacteria (LAB) are safe for human consumption and beneficial to human health when used as probiotics. Clinically, they can improve digestive health and have various medical applications (Holzapfel and Wood, 2014). For instance, *Limosilactobacillus reuteri* DSM 17938 helps to prevent and alleviate antibiotic-associated diarrhea (Kołodziej and Szajewska, 2019). *Lactobacillus sakei* CRL1862 can control biofilms from *Listeria monocytogenes* by producing bacteriocin (Pérez-Ibarreche et al., 2016). As common inhabitants of the gastrointestinal tract (Salas-Jara et al., 2016), *Lactobacillus* spp. have been used in genetic and molecular biology studies for heterologous expression, demonstrating their important roles in investigating the properties of probiotics and their potential as live *in vivo* carriers (Wyszyńska et al., 2015). A previous study showed that recombinant invasive *Lactiplantibacillus plantarum* expressing fibronectin binding protein A specifically modulated humoral immune responses (Liu et al., 2019). Alimolaei et al. (2017) found that *Lcb. casei* harboring the α-toxoid from *Clostridium perfringens*, as an oral vaccine candidate, was able to protect the host from gas gangrene and necrotic enteritis. However, the specific interaction mechanisms between LAB and their hosts remain unknown. In our previous study, we suggested that bile salt hydrolase (BSH), as a functional enzyme in LAB, is related to the intestinal colonization of LAB (Yang et al., 2019). To further study the probiotic mechanism of LAB carrying *bsh in vivo*, a stable and specific microbial tracer technology is necessary for monitoring the *in vivo* distribution, colonization, and survival of LAB.

Fluorescent proteins (FPs) are stable and species spanning and thus have served as intracellular biochemical markers for more than two decades (Zhang and Xing, 2010). Labeling probiotics with FPs is an effective strategy for tracing target cells in complex *in vivo* ecosystems and evaluating *in vitro* gene expression (Landete et al., 2016). Among them, mTagBFP2, mCherry, and green fluorescent protein (GFP) have been expressed in LAB (Landete et al., 2016; Spacova et al., 2018). The adhesion of *Lacticaseibacillus rhamnosus* to Caco-2 cells was visualized with mTagBFP2 and mCherry (Spacova et al., 2018). The activities of the *Lpb. plantarum* strain B35 labeled with GFP were monitored in the digestive tract of honey bees (Javorský et al., 2017). In our previous study, the fluorescence emitted from eGFP protein in *Lpb. plantarum* WCFS1 did not distinguish the background and host cells, whereas mCherry protein displayed prominent fluorescence activity without interference (Chen et al., 2019), indicating that mCherry is more suitable for tracing *Lpb. plantarum* WCFS1. Moreover, mCherry protein does not exert toxic or additional physiological effects on host organisms (Carroll et al., 2010).

Most fluorescent markers rely on plasmid expression systems, in which antibiotic selection is required (Landete et al., 2016). In other words, when the selection pressure is lost, the number of cells maintaining the plasmids tends to decrease during subculture. Segregational stability studies estimated that only 3% of recombinant pSIP-series vectors are retained after 84 generations without antibiotic selection (Nguyen et al., 2011). Therefore, integrating the genes encoding FPs into probiotic genomes is a better strategy for ensuring the expression stability of exotic FPs in the absence of antibiotics, especially for tracking targets *in vivo*.

In our previous study aimed at establishing an appropriate FP labeling technology, we discovered that the constitutive promoter P_*ldhL*_ was optimal for mCherry expression in WCFS1 (Chen et al., 2019), as it does not require additional inducers and makes the recombinant bacteria useful for *in vivo* tracing research. In the same study, we labeled *Lpb. plantarum* with FPs by constructing and introducing a plasmid constitutively expressing mCherry (Chen et al., 2019).

In the present study, we describe the construction of a novel mCherry labeling system, the encoding gene of which was integrated into the bacterial chromosome *via* double-crossed homologous recombination. The stability of the new mCherry labeling system was observed and compared to the previous one harboring a plasmid. Furthermore, the research application of the integrated mCherry labeling system in studying LAB *in vivo* adhesion and colonization was tested by visualizing and quantifying the *in vivo* colonies of the strain carrying *bsh*, which encodes the enzyme that promotes LAB adhesion to Caco-2 cells (Yang et al., 2019).

## Materials and Methods

### Bacterial Strains and Culture Conditions

The bacterial strains used in this study are listed in Table 1. All the *Lpb. plantarum* WCFS1 and derivative strains used in this study were cultivated in MRS broth (Haibo Ltd., Qingdao, China) at 37°C with erythromycin (Em), chloramphenicol (Cm), and rifampicin (Rm) at a final concentration of 10, 10, and 50 μg ml^–1^ when required, respectively. *Escherichia coli* DH10B was used for cloning purposes, and it was cultivated at 37°C in Luria-Bertani (LB) medium (Oxoid Ltd., Basingstoke Hampshire, United Kingdom) with Em, Cm, and ampicillin (Amp) at a final concentration of 250, 10, and 100 μg ml^–1^ when required, respectively.

**TABLE 1:** Strains and plasmids used in this study.

Strains or plasmids	Relevant characteristics^†^	References
**Strains**		
*Lactiplantibacillus plantarum*		
WCFS1	Wild type	Kleerebezem et al., 2003
NZ5327	Cm^*r*^, containing a *lox66*-P_32_-*cat-lox71*-P_*ldhL*_-*mcherry* cassette stably integrated into the downstream of Rm1 locus	This study
NZ5328	Derivative of NZ5347, containing a *lox72*-P_*ldhL*_-*mcherry* cassette with the removal of *cat* gene	This study
*Escherichia coli*		
DH10B	Cloning host	Durfee et al., 2008
**Plasmids**		
pNZ5319	Cm^*r*^, Em^*r*^, containing a *lox66*-P_32_-*cat-lox71* fragment	Lambert et al., 2007
pNZ5348	Em^*r*^, Cre-recombinase expression	Lambert et al., 2007
pNZ5328	Cm^*r*^, Em^*r*^, pNZ5319 derivative containing homologous regions up- and downstream of the inserted site and a P_*ldhL*_-*mcherry* fragment	This study
pmCherry-C1	Kan^*r*^, containing *mcherry* gene	Taylor et al., 2011
pSIPH460	Em^*r*^, pSIP403 derivative, *gusA* replaced by a multiple cloning site containing two his-tag fragments	Chen et al., 2018
pSIPH472	Em^*r*^, pSIPH460 derivative containing *mcherry* gene	Chen et al., 2019
pSIPH462	Em^*r*^, pSIPH460 derivative containing *bsh* gene of *L. reuteri*	Yang et al., 2019
pLDHLH673	Em^*r*^, pSIPH460 derivative, the promoter P_*ldhL*_ of the *ldhL* gene was retained and the original start codon of *mcherry* was replaced from TTG to ATG.	This study
pLDHLH674	Em^*r*^, pSIPH460 derivative, six bases CTTATT upstream of the ATG start codon of *mcherry* was replaced by a *Bam*HI site	This study
pLDHLH675	Em^*r*^, pSIPH460 derivative, the spacer between SD region, and the start codon of the *mcherry* gene contained the 15 nucleotides coding for the first five amino acids of *ldhL* but lacking the ATG start codon of *mcherry*	This study

*^†^Kan^r^, kanamycin resistant; Em^r^, erythromycin resistant; Cm^r^, chloromycetin resistant.*

### General DNA Techniques

Genomic DNAs of WCFS1 were extracted according to the instructions provided in the Rapid Bacterial Genomic DNA Isolation Kit (Sangon, Shanghai, China). All plasmids used in this study were extracted using AxyPrep Plasmid MiniPrep Kit (Axygen, CA, United States) according to the manufacturer’s protocol. Enzymes were purchased from Takara (Dalian, China). PCRs for cloning purposes were performed with *Ex*Taq PCR DNA polymerase (Takara, Dalian, China) following the manufacturer’s instructions. All amplified sequences were verified and the nucleotide sequencing in this study was performed at Sangon (Shanghai, China).

### Optimization of the Promoter Spacer for mCherry Expression

**FIGURE 1:**
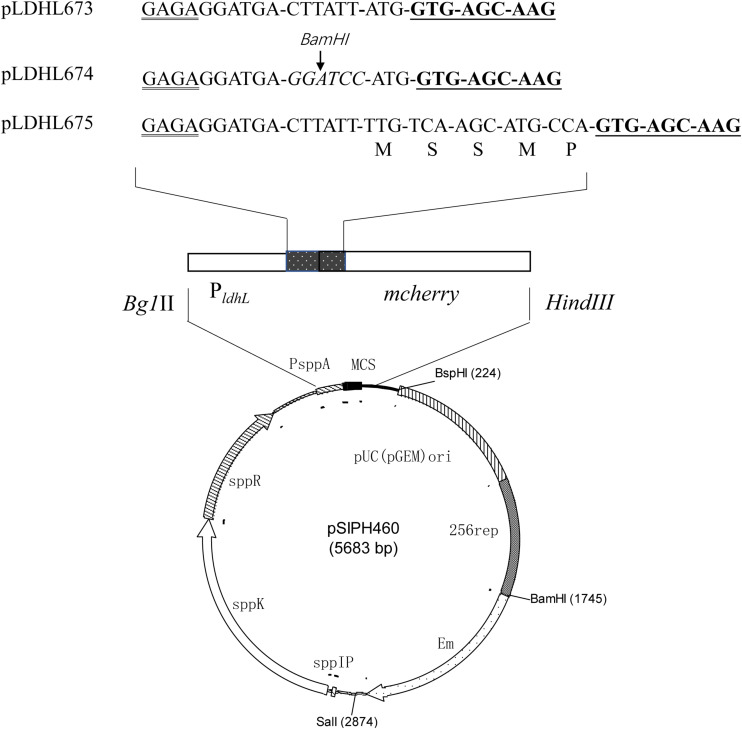
Optimization of the promoter spacer for mCherry expression. The Shine–Dalgarno (SD) sequence is doubly underlined and the *mcherry* gene is underlined. The spacer, which is between the SD sequence of the promoter P_*ldhL*_ and the start codon in *mcherry*-containing expression vectors, was designed variously. The amino acid residues, as the restriction enzyme recognition sites, are marked with italics.

To optimize the expression of the *mcherry* gene, three different plasmids, pLDHL673, pLDHL674, and pLDHL675, were constructed, respectively. All these three plasmids were, respectively, constructed by cloning a P_*ldhL*_-*mcherry* gene cassette into the *Bgl*II–*Hin*dIII site of plasmid pSIPH460 (Figure 1), in which the different P_*ldhL*_-*mcherry* gene cassette was constructed based on the previous literature method (Kahala and Palva, 1999; Anbazhagan et al., 2013; van Zyl et al., 2015). In pLDHL673, the promoter P_*ldhL*_ of the *ldhL* gene was retained and the original start codon of *mcherry* was replaced from TTG to ATG. The spacer of pLDHL673 between the Shine-Dalgarno (SD) sequence of the promoter P_*ldhL*_ and the start codon of *mcherry* was 12 bases. In pLDHL674 (van Zyl et al., 2015), the spacer between the SD region of the promoter P_*ldhL*_ and the ATG start codon of *mcherry* was also 12 bases; however, six bases CTTATT upstream of the ATG start codon of *mcherry* was replaced by a *Bam*HI site. Similarly, in pLDHL675 (Kahala and Palva, 1999), the spacer between the SD region and the start codon of the *mcherry* gene contained the 15 nucleotides coding for the first five amino acids of *ldhL* but lacking the ATG start codon of *mcherry*. All these three P_*ldhL*_-*mcherry* fragments were oligonucleotides synthesized by GenScript (Nanjing, China) with the *Bgl*II and *Hin*dIII sites at its 5′ and 3′ ends, respectively, cloned and introduced to *E. coli* DH10B. Positive colonies were selected on plate assays. After confirming the correctness of the plasmid constructs, the resulting expression vectors were designated as pLDHL673, pLDHL674, and pLDHL675, respectively. Subsequently, plasmids pLDHL673, pLDHL674, and pLDHL675 were, respectively, electroporated in WCFS1, and transformants were screened by selecting for 250 μg ml^–1^ of Em resistant. Resulting strains were designated as WCFS1:pLDHLH673, WCFS1:pLDHLH674, and WCFS1:pLDHLH675, respectively.

### Fluorescence Detection of mCherry in Recombinant WCFS1

Recombinant WCFS1 carrying different expression vectors were, respectively, grown in MRS broth for 12 h with 10 μg ml^–1^ of Em. It is worth noting that WCFS1:pSIPH472 needs to be induced by adding SppIP as an inducer peptide at a final concentration of 50 ng L^–1^ and incubated at 30°C as previously described (Chen et al., 2019). The 0.5-ml cultures were then harvested (8,000 × *g*, 3 min), and the cells were washed twice with PBS (pH 7.0) to remove culture medium and then resuspended in 0.5 ml of PBS (pH 7.0). The fluorescent intensity of the cells was detected with Multiscan Spectrum (Tecan, Swiss) at an excitation of 587 nm and an emission of 612 nm, respectively. In each assay, the strain carrying pSIPH460 was used as the control.

### Construction of the Genomic Integrated mCherry Mutants

**FIGURE 2:**
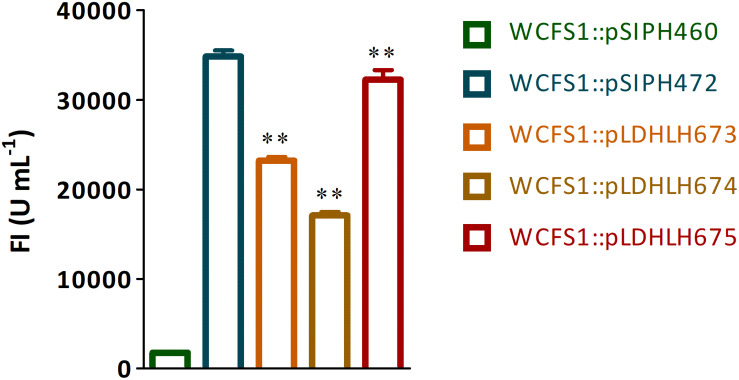
Fluorescence detection of mCherry protein expressed in recombinant WCFS1. The fluorescence intensity of recombinants WCFS1 containing the different mCherry expression vectors were detected at the corresponding wavelength, respectively. The pLDHLH-series plasmids and pSIPH472 plasmid all carry the target *mcherry* gene, while the pSIPH460 plasmid is set as the control. ** Represents a significant (*p* < 0.01) difference between groups.

Expression and integration plasmids constructed during this study are based on the pNZ5319 LAB/*E. coli* shuttle vector (Rolain et al., 2012). The vector contains an *Em*^*R*^ gene for Em resistance, the promoter P_32_, and a *cat* gene for Cm resistance. All primers are listed in Table 2. The targets used for homologous recombination were the restriction modification type 1 (*Rm1*) gene for WCFS1 (Serrano et al., 2007). The double-crossover integration vector was constructed in such a way that the *lox66*-P_32_-*cat-lox71*-P_*ldhL*_-*mcherry* gene cassette (Figure 2) was flanked with the target genes’ sequences including some of their upstream and downstream regions, to facilitate homologous recombination.

**TABLE 2:** PCR and quantitative PCR primers used in this study.

Primer	Sequence (5′ to 3′)^†^	References
**PCR**		
PF	*AGATCTGAGCTC*AATCTTCTCACCGTCTTG	This study
PR	*CATATG*TCAATAAGTCATCCTCTCGTAG	This study
mR	*AAGCTT*TCAGTGGTGGTGGTGGTGGTGCTTGTACAGCTCGTCCATG	This study
21	TCCTCGCCCTTGCTCACCATTC*CCATGG*AATAAGTCATCCTCTCGTAG	This study
22	CTACGAGAGGATGACTTATT*CCATGG*GAATGGTGAGCAAGGGCGAGGA	This study
211	*CTCGAG*CTCCTGCATATCTGCAGAAC	This study
212	*ATTTAAAT*GTCGACCAGAATATTATTCGC	This study
221	*AAGCTT*TTACCACGCATCAATTTGAGCG	This study
222	*AAGCTTAGATCT*CCGTTTCTTACGTGGATGCG	This study
cexu4	CAGTGGAACGAAAACTCACG	This study
cexu5	GTGAATCATAGGTGGTATAG	This study
SW	ACCAGCCGTTATCACTAATG	This study
85	GTTTTTTTCTAGTCCAAGCTCACA	Lambert et al., 2007
XW	CGTGACTTGAACTTAACGC	This study
EryintF	CGATACCGTTTACGAAATTGG	Lambert et al., 2007
EryintR	CTTGCTCATAAGTAACGGTAC	Lambert et al., 2007
Cat96F	TCAAATACAGCTTTTAGAACTGG	Lambert et al., 2007
Cat97R	ACCATCAAAAATTGTATAAAGTGGC	Lambert et al., 2007
**qPCR**		
Qm1	TTCATGTACGGCTCCAAGGC	This study
Qm2	GTCCTCGAAGTTCATCACGC	This study
rpoBF	CACCGTACCCGTAGAAGTTATGC	Marco et al., 2007
rpoBR	GGAGACCTTCATCCAAGAACCA	Marco et al., 2007

*^†^Restriction sites are underlined and italic.*

The upstream sequence (primers 211 and 212, 900 bp, Table 2) and the downstream sequence (primers 221 and 222, 900 bp, Table 2) of the *Rm1* gene were amplified from the WCFS1 genome DNAs, respectively. The downstream fragment was digested with *Hin*dIII and then ligated to the LAB/*E. coli* shuttle vector pLDHLH675 that contains the P_*ldhL*_-*mcherry* gene and was digested with the same restriction enzyme. The presence of target genes in the resulting recombinant plasmid pLDHLH675-Rmdown isolated from Em-resistant transformants was confirmed by sequencing with primers 221/222. Plasmid pLDHLH675-Rmdown was digested with *Bg1*II and *Sac*I, and a 2.2-kb DNA fragment, containing the *mcherry* gene, flanked by the P_*ldhL*_ promoter and the downstream regions, was purified from a 1% (w/v) agarose gel. The upstream fragment was digested with *Xho*I and *Swa*I and then ligated to plasmid pNZ5319, carrying the *lox66*-P_32_-*cat-lox71* gene cassette, which was digested with the same restriction enzyme. Resulting recombinant plasmid pNZ5319-Rmup was confirmed by sequencing with primers 211/212. Plasmid pNZ5319-Rmup was linearized following digestion with *Bg1*II and *Sac*I, purified, and then ligated with T_4_ DNA ligase to the 2.2-kb P_*ldhL*_-*mcherry-Rmdown* gene fragment. *E. coli* DH10B electro-cells were then transformed for propagating the resulting plasmid, named pNZ5328. Transformants were screened by selecting for Cm and Em resistant and a red colony color phenotype. The integrity of the plasmid was verified by restriction digests and PCR with primers 211 and 222. Following sequence confirmation of the integration vectors, pNZ5328 was extracted and transformed into WCFS1 by electroporation as described previously (Lambert et al., 2007). The Cm marker was associated with the *mcherry* gene to enable selection of integrants that have lost the *Em*^*R*^-associated vector. The *lox66*-P_32_-*cat-lox71*-P_*ldhL*_-*mcherry* gene was verified by PCR with primers SW/85 and PF/XW, respectively (Table 2). The resulting mutant was named NZ5328.

### Stability of the Integrated mCherry Expression

The integrated mCherry mutant NZ5328, with WCFS1:pLDHLH675 as a control, were continuously subcultured in MRS medium without antibiotics for 100 generations, respectively. Subsequently, the cell numbers of these two strains were counted in the corresponding MRS agar, with or without antibiotic, after 100 generations by replica plating, respectively (Nguyen et al., 2011). All colonies in MRS agar with antibiotics were re-verified through high-throughput PCR to detection of the *mcherry* gene using primers Qm1 and Qm2 (Table 2).

### Growth Curve of the Integrated mCherry Mutant

The integrated mCherry mutant NZ5328, with WCFS1:pLDHLH675 as control, were cultivated in MRS broth overnight at 37°C, respectively. The growth curve of the strain was performed by measuring OD_600 *nm*_ value of bacterial solution, and the corresponding fluorescent intensity was also measured as previously described in this study. Meanwhile, the wild-type WCFS1 was set as a negative control.

### Enumeration for Viable Cells of the Integrated mCherry Mutant

The integrated mCherry mutant NZ5328 was cultivated in MRS broth for 10 h at 37°C. After then, the culture was serially diluted, and the fluorescence of each cell suspension was measured as previously described in this study. Cell suspensions were plated onto MRS agar, and the plates were incubated at 37°C for 48 h. Fluorescence emitted was expressed as fluorescent intensity against viable cell numbers.

In addition, cell suspensions (1 ml) of the 10-h-old cultures NZ5328 were harvested. The cell suspensions were disrupted by glass beads (0.1 mm, biospec). Genome DNAs of viable cells were then extracted and serially diluted 10-fold, and 1 μl of each dilution was subjected to the qPCR assay on an ABI 7500 Fast real-time PCR system with 2.0.1 version software. The reaction mix contained 1.0 μl of DNA, 0.3 μM each of *mcherry*-specific primers (Qm1 and Qm2, Table 2), and 10 μl of 2×TaqMan Universal PCR Master Mix (TaKaRa Ltd., Dalian, China). The thermal cycle program was as follows: 95°C for 30 s, 95°C for 5 s, followed by 40 cycles of 58°C for 30 s and 72°C for 30 s. Meanwhile, the *rpoB* gene from the WCFS1 genome was used as a reference gene as well as the control (Marco et al., 2007).

### Fluorescence Detection of WCFS1 Mutants in Mice Gut

The integrated mCherry mutant NZ5328 and the wild-type WCFS1 were cultivated in MRS medium overnight, and harvested by centrifugation (8,000 × *g*, 3 min), respectively. These two kinds of cells were washed twice and re-suspended with 1 ml of PBS (pH 7.0). In total, 14 healthy mice (C57BL/6, 8 weeks old) were raised for tracing experiment and divided into two groups (seven mice per group). Each group was fed with 0.2 ml of bacterial suspensions (NZ5328 or WCFS1, 10^9^ cells) daily for two consecutive days, respectively. Moreover, one mouse in each group was randomly selected and euthanized with ketamine injection, after feeding the bacterial suspensions for 6 h. The intestinal tissues of these two mice were used for fluorescence imaging and observed by the *in vivo* imaging system (IVIS, PerkinElmer, Massachusetts, United States). Ethical approval for animal experiments was obtained from the Animal Research Ethics Committee of Nanjing Normal University (Permit No. AREC 2018-10-26). All animal procedures were performed according to European Community guidelines (Directive 2010/63/EU).

### *In vitro* Bacterial Adhesion Assay

Previously, the results of the plate colony counting assay proved that BSH protein promotes the adhesion of LAB to Caco-2 cells (Yang et al., 2019). In this study, the same expression vector pSIPH462 was cloned into mCherry labeled NZ5328 to study the change of its adhesion characteristics. After electrotransformation of the plasmid pSIPH462 carrying the *bsh* gene into NZ5328, the strain NZ5328 carrying pSIPH462 (named NZ5328-*bsh*) was cultivated in MRS medium with 10 μg ml^–1^ of Em and confirmed by PCR using primer cexu4 and cexu5 (Table 2). The recombinant strain was cultured in MRS medium supplemented with SppIP and until the OD_600 *nm*_ reached about 1.8, the BSH activities of NZ5328-*bsh* or WCFS1:pSIPH462 (named WCFS1-*bsh*) were detected as described previously, respectively (Yang et al., 2019). The recombinant strain NZ5328:pSIPH460 or WCFS1:pSIPH460 was set as an experimental control. Subsequently, the adhesion of NZ5328-*bsh* strain to Caco-2 cells was performed as described by Yang et al. (2019). The total number of adhered BSH recombinant cells was estimated by plate count in MRS agar, fluorescent assay, and *mcherry*-qPCR, respectively. The adhesion ability (CFU/cell) was based on the ratio of adhered BSH recombinant cells to total Caco-2 cells, and the adhered BSH recombinant cells were observed under the confocal laser scanning microscope (CLSM, Olympus, Tokyo, Japan). The recombinant strain NZ5328:pSIPH460 was also set as an empty control, and each sample was repeated at least three times.

### Statistical Analysis

The values in this study were presented as mean ± standard deviation (SD). Statistical analyses are conducted by GraphPad prism 6.0 (GraphPad Software Inc., San Diego, CA, United States). Statistical comparisons are analyzed by Student’s *t*-test, and multiple comparisons are performed with one-way ANOVA analysis, followed by Turkey’s *post-hoc* test. A *p*-value of less than 0.05 was considered as statistically significant, and ^∗^*p* < 0.05, ^∗∗^*p* < 0.01.

## Results

### Optimization Promoter Spacer for mCherry Expression in WCFS1

To optimize the expression of mCherry controlled by the promoter of *ldhL* in WCFS1, a series of mCherry expression vectors was constructed by adjusting the spacer between the SD region and start codon of *mcherry* (Figure 1). Three different recombinant plasmids (pLDHLH674, pLDHLH675, and pLDHLH673) were, respectively, constructed and electrotransformed into the host strain WCFS1. The expression of *mcherry* was evaluated by detecting its fluorescence intensity. The results showed that the fluorescent mCherry proteins were successfully expressed in the three recombinant strains (Figure 2). The fluorescence intensity of strain WCFS1:pLDHLH675 (32254.25 U ml^–1^) was higher than that of strains WCFS1:pLDHLH673 (23217.5 U ml^–1^) and WCFS1:pLDHLH674 (17085.25 U ml^–1^) (Figure 2), indicating that the promoter spacer of pLDHLH675 was more suitable for expressing the fluorescent mCherry protein. Finally, we determined the optimized spacer structure of pLDHLH675, which was used for subsequent mCherry expression in this study.

### Construction of Genomic-Integrated mCherry Mutants of WCFS1

**FIGURE 3:**
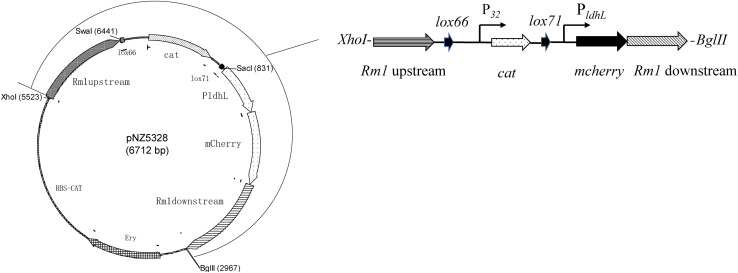
Schematic overview of exogenous gene insertion in pNZ5328 vector. Vector pNZ5328 contains *Rm1* gene upstream, a *lox66* gene, the promoter P_32_, a *cat* gene, a *lox71* gene, the promoter P_*ldhL*_, the *mcherry* gene, and *Rm1* gene downstream.

The double-crossover integrated mCherry expression plasmid pNZ5328 was constructed as described previously (Figure 3). *Escherichia coli* DH10B cells transformed with pNZ5328 produced fluorescent colonies that were both Em- and Cm-resistant. NZ5328 retained resistance to Cm, but not to Em, indicating that the *lox66*-P_32_-*cat-lox71*-P_*ldhL*_-*mcherry* cassette was successfully integrated by double-crossover into the host chromosome without the plasmid DNA encoding *Em*^*R*^ (Figure 3). The presence of *cat* and *mcherry* in NZ5328 was confirmed using polymerase chain reaction (PCR) with the primers SW/85 and PF/XW, respectively. No 4.2-kb target PCR fragment was amplified, confirming that *Rm1* was successfully disrupted by the insertion of the *lox66*-P_32_-*cat-lox71*-P_*ldhL*_-*mcherry* cassette. The results of fluorescence detection showed that NZ5328 was fluorescent with an intensity of 26,394 U ml^–1^, indicating that the genomic-integrated strain for expressing mCherry was successfully constructed.

### Stability of the Integrated mCherry Expression System in WCFS1

**FIGURE 4:**
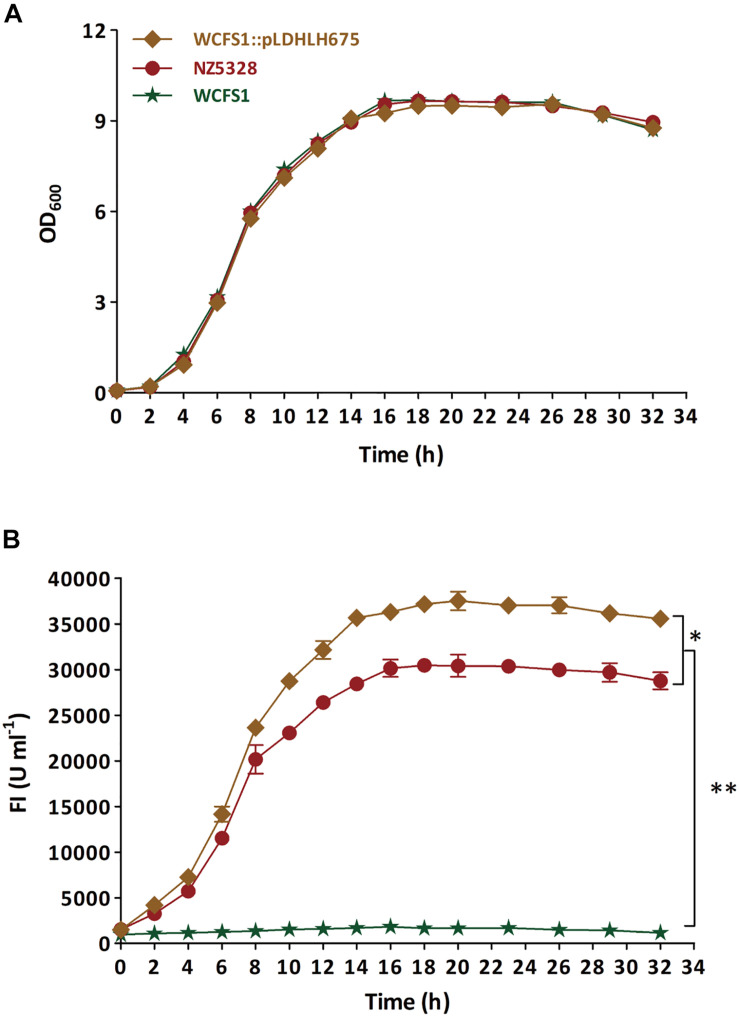
The growth curve **(A)** of recombinants carrying *mcherry* gene and detection of corresponding fluorescence values **(B)**. The WCFS1:Pldhlh675 is plasmid-based recombinant, and the NZ5328 is genomic-integrated recombinant, while the wild-type WCFS1 is set as the control. * Represents a significant (*p* < 0.05) difference between groups. ** Represents a significant (*p* < 0.01) difference between groups.

To verify the stability of the integrated mCherry expression system in NZ5328, the target *mcherry* and fluorescence intensity of the system were detected and evaluated. First, the growth curve of NZ5328 was measured, and its corresponding fluorescence intensity was detected (Figure 4). Compared to those of the control strain WCFS1:pLDHLH675 and wild-type WCFS1, there was no difference in the growth curves of the three strains (Figure 4A). Compared to that of the control strain WCFS1:pLDHLH675, the fluorescence intensity of the integrated strain NZ5328 decreased slightly (*p* < 0.05) but tended to be stable at the stationary stage (maximum fluorescence: 30,447.33 ± 959.37 U ml^–1^; Figure 4B).

**FIGURE 5:**
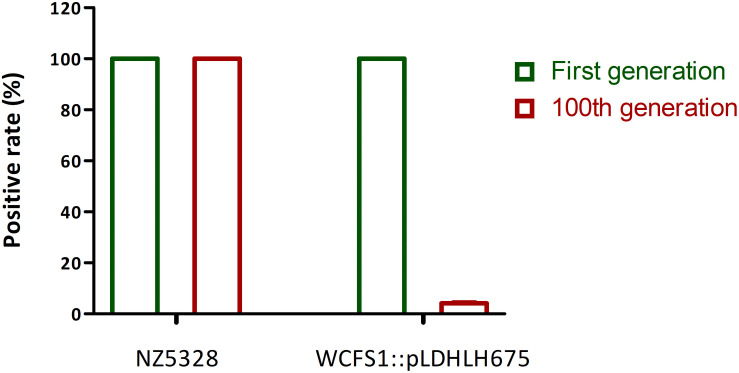
Stability of *mcherry* gene in genomic-integrated and plasmid-based recombinants (after 100 generations). The left *Y*-axis is the percentage of *mcherry*-containing (positive) colonies in the MRS agar with antibiotic.

After 100 generations, both replica plating and PCR verification results showed that the integrated strain NZ5328 stably carried *mcherry* without loss (Figure 5, PCR validation data not shown), and the fluorescence intensity of the NZ5328 derivatives was maintained at a high level (32,164.25 ± 128.12 U ml^–1^). However, the WCFS1:pLDHLH675 derivatives exhibited significant *mcherry* gene loss. Almost 95% of WCFS1:pLDHLH675 derivatives could not grow on plates containing antibiotics (Figure 5). The results showed that plasmids were easily lost owing to the lack of resistant stress selection.

### Enumeration of Viable Cells With the Integrated mCherry Mutant

**FIGURE 6:**
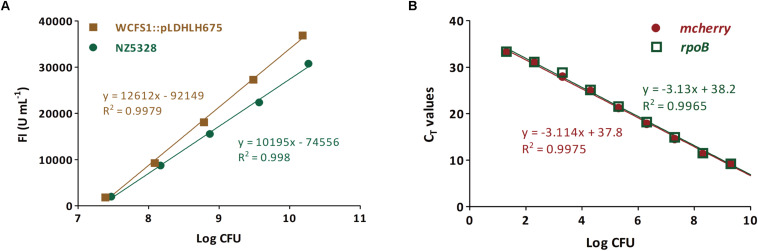
Enumeration for viable cells of the *mcherry*-containing mutants (genomic-integrated and plasmid-based) based on the fluorescence intensity **(A)** and *mcherry*-qPCR **(B)**. The *rpoB* gene from NZ5328 genome was used as an internal gene.

To verify whether the integrated mCherry expression system can be used for the quantitative detection of the target bacteria, the correlation between fluorescence intensity and cell numbers of the integrated mCherry mutant was investigated. As expected, the change in the trend of cell numbers was similar to that of the fluorescence intensity of *mcherry*-containing strains over time. There was a linear relationship between the fluorescence intensity and number of cells in NZ5328 (*R*^2^ = 0.998; Figure 6A), indicating that fluorescence detection can be used for enumeration of cells.

Subsequently, to confirm that *mcherry* in the integrated mutant can be used as the target gene for quantification, a pair of specific primers for *mcherry* was developed for enumerating cells. The quantitative (q)PCR assay was positive for *mcherry*, and no cross-reaction with non-target genes was observed. Moreover, *mcherry* quantification was stable and highly reproducible (data not shown). To further verify the quantitative accuracy, *mcherry* in NZ5328 was evaluated using the reference gene *rpoB* (Marco et al., 2007). The standard curve based on *rpoB* almost overlapped with that of *mcherry*, with a correlation coefficient of 0.9965 (Figure 6B), suggesting that quantification of *mcherry* in NZ5328 was consistent with that of *rpoB*. Thus, the qPCR assay based on *mcherry* detection can be applied to measure viable counts over a dynamic range from 10 to 10^10^ colony-forming units (CFU).

### Fluorescence Localization and Detection of Target Bacteria in Mouse Intestines

**FIGURE 7:**
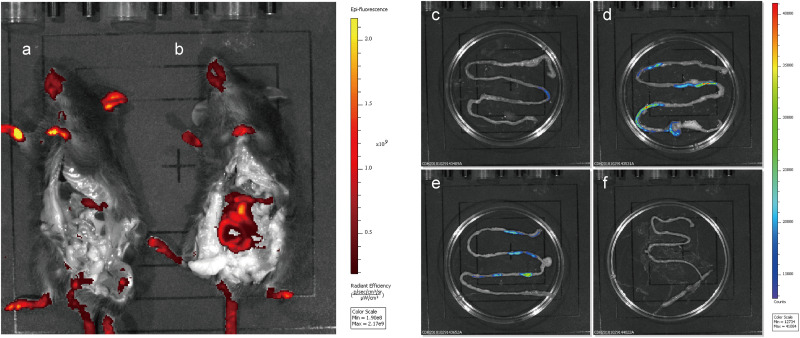
Fluorescence imaging and tracing of WCFS1 and derivative cells in the abdomen and small intestine of the C57BL/6 mice. After 6 h of feeding, euthanized mice were treated with the removal of ventral skin when fluorescence imaging was collected. Fluorescence determination of the C57BL/6 mouse fed WCFS1 **(a)** and NZ5328 **(b)** by the *in vivo* imaging system, and fluorescence detection of WCFS1 **(f)** and NZ5328 **(c–e)** in the small intestine of the C57BL/6 mouse.

To evaluate the fluorescence of the integrated mCherry mutant in the mouse gut without interference from the host, mice were fed integrated mCherry mutants, and red fluorescence was monitored. Fluorescence was detected in the gastrointestinal tract of the mice after removing the abdominal skin and fur (Figure 7), and only the limbs and tails of the mice presented red fluorescence at the emission wavelength of mCherry (data not shown). At 6 h after NZ5328 administration, the persistence of fluorescent cells in the small intestine was perceived when the mice were imaged (Figure 7). Fluorescence was not emitted by WCFS1 or the intestinal tissues, indicating that the background without mCherry protein did not interfere with fluorescence determination at the corresponding wavelength.

### Verification of Integrated Bacteria in Cell Adhesion, Quantification, and Protein Expression

**FIGURE 8:**
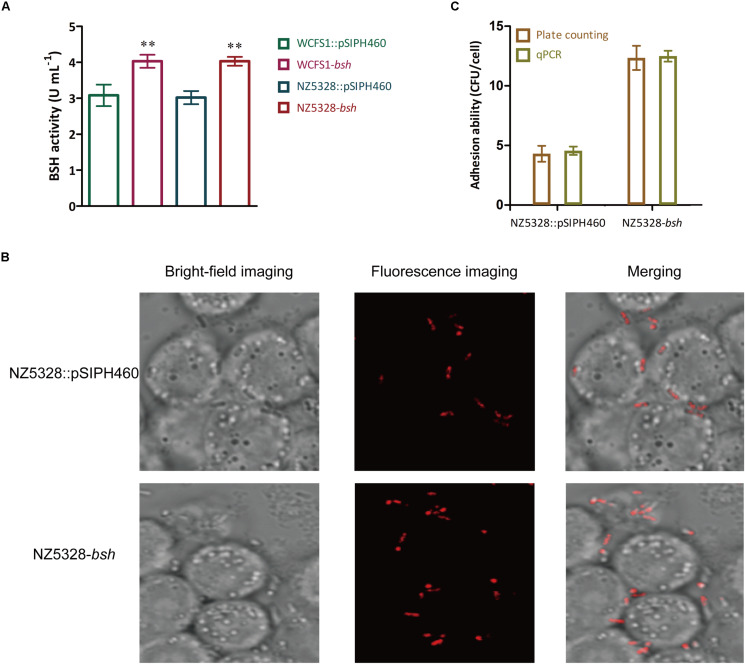
BSH activity detection **(A)**, fluorescence imaging **(B)**, and quantitative detection **(C)** of the bacterial adhesion assay *in vitro*. ** Represents a significant (*p* < 0.01) difference from the control.

BSH activity determined in BSH recombinants showed that NZ5328-*bsh* expressing heterogenous *bsh* was not distinct from that of WCFS1 harboring pSIPH462 (Figure 8A), suggesting that the chromosomal-integrated FP gene did not interfere with the heterologous expression of BSH protein in the WCFS1 strain. These results provide a theoretical basis for NZ5328 to be used as an expression host for other target genes. In this study, with the assistance of mCherry labeling, changes in the adhesion of LAB promoted by BSH proteins could be directly observed through microscopes. After co-culture with Caco-2 cells, BSH recombinant cells were visualized using confocal laser scanning microscopy as red fluorescence emitted by mCherry (Figure 8B). Furthermore, to complement the approach using fluorescent signals to quantify the LAB cells, qPCR analysis of the *mcherry* gene was performed to quantify LAB cells. The results obtained using *mcherry*-qPCR and plate counts were similar (Figure 8C). The approach used in this study has fewer steps and higher efficiency than the plate colony counting assay, and the results were comparable between them.

## Discussion

In this study, we aimed to construct a stable red fluorescent protein (RFP) mCherry expression system in WCFS1 and found that the chromosomal-integrated mCherry labeling system can be extensively used to examine the distribution, colonization, and survival of LAB *in vivo*.

Previous studies have suggested that photons emitted by the mCherry protein reflect the *in vivo* metabolic activity of *L. sakei* and *Lpb. plantarum* 423 (van Zyl et al., 2015). Our previous studies also suggested that the RFP mCherry has potential applications in the labeling of WCFS1 (Chen et al., 2019). The mCherry protein can be constitutively expressed under regulation of the promoter P_*ldhL*_. In our previous study, the fluorescence intensity emitted by mCherry regulated by the promoter P_*ldhL*_ in WCFS1:pLDHLH673 was visible but significantly lower than the induced expression (Chen et al., 2019). The spacer region modified by embedding simple sequence repeats affected the expression of target genes (Egbert and Klavins, 2012). In this study, we first optimized the mCherry expression level by changing the spacer size. Compared to that of the inducible strain WCFS1:pSIPH472, the fluorescence intensity of WCFS1 harboring pLDHLH674 and pLDHLH673 was markedly lower than that of WCFS1:pSIPH472 (Figure 2), which stimulated intense fluorescence activity as described previously (Chen et al., 2019). In contrast, we found that the insertion of restriction enzyme recognition sequences into the space between the SD region and start codon altered the mCherry expression level. Fluorescence emission by mCherry in WCFS1:pLDHLH675 reached a maximum (Figure 2), indicating that the 9-bp spacer was optimal for higher protein expression.

Among the fluorescent markers used in LAB, GFP, RFP, and their derivatives are commonly employed (Landete et al., 2016; Javorský et al., 2017; Spacova et al., 2018). Cellular auto-fluorescence emission of WCFS1 has an intensive wavelength overlap with that of eGFP (Chen et al., 2019). In contrast, mCherry produced clear red fluorescence without background interference in WCFS1, which is consistent with RFP performance in other *Lactobacillus* spp. (van Zyl et al., 2015; Spacova et al., 2018). Notably, the *Lpb. plantarum* strain labeled with mCherry was not pink under natural light, unlike the color of other mCherry-labeled bacteria in previous research (Castro-Bravo et al., 2017; Martinez-Jaramillo et al., 2017; Mohedano et al., 2019); we speculate that the unique cell wall structure of WCFS1 may be the main reason.

Most fluorescence reporter systems are dependent on expression plasmids carrying a fluorescence gene. The segregational stability of plasmids varies with selection pressure. Without antibiotics, expression vectors can be easily lost from the host cells during continuous proliferation, particularly *in vivo*. Integration of *mcherry* into the genome of a probiotic strain not only excludes the need for antibiotic selection but also provides an expression system that is genetically stable. When *mcherry* was integrated into WCFS1, the level of fluorescence emitted from labeled cells decreased compared to that observed in plasmids (Figure 4B). This may have resulted from the low transcriptional abundance of the insertion locus. Genetic stability was high, although the fluorescence intensity of *mcherry*-integrated cells was low (Figure 4B). After 100 generations, the integrated strain NZ5328 stably carried *mcherry* without loss (Figure 5) and fluorescence emitted from the labeled strains remained constant (data not shown), reflecting the superiority of the fluorescence integration strategy. Thus, fluorescence genes integrated into chromosomes can be genetically stable and used to track each step of cell movement in real time, while eliminating the potential risk of plasmid loss.

In addition to tracing, the integrated mCherry expression system shows potential for enumeration. *mcherry-*labeled cells were determined based on standard fluorescence curve intensity and threshold cycle (C_*T*_) values for *mcherry*. In our study, a positive linear correlation between fluorescence intensity and CFU was successfully established (Figure 6). The number of *Lpb. plantarum* cells carrying integrated *mcherry* positively correlated with fluorescence intensity, which is in agreement with the findings of a colonization study (van Zyl et al., 2015). However, considering the limited detection sensitivity, the intensity of fluorescent signals cannot reflect the number of cells when <10^7^ CFU. Therefore, we used qPCR to quantify *mcherry* in the NZ5328 genome as an alternative. The relationship between the cell numbers and C_*T*_ values of amplified *mcherry* was linear over a broad dynamic range, and starting material as low as 10 CFU could still be detected. The two quantitative methods facilitated rapid detection of target strains *in vivo* and *in vitro*, and considerably improved work efficiency.

In addition to the extensive studies on LAB as probiotics, they have also been intensively utilized as the host strains for *in vivo* heterologous expression of many important proteins, along with the improvement of LAB expression systems. In this study, *bsh* gene was expressed in the mCherry-labeled WCFS1 strain. The results suggested that the chromosomal integrated FP gene did not interfere with the heterologous expression of BSH protein in the WCFS1 strain. Previously, the results of the plate colony counting assay proved that BSH protein promotes the adhesion of LAB to Caco-2 cells (Yang et al., 2019). In this study, with the assistance of mCherry labeling, changes in the adhesion of LAB promoted by BSH proteins could be directly observed through microscopes (Figure 8B). In order to complement the approach using fluorescence signals to quantify LAB cells, qPCR analysis of the *mcherry* gene was performed to further quantify the LAB cells.

Nowadays, *Lactobacillus* spp. have been extensively studied not only for their probiotic properties (Holzapfel and Wood, 2014), but also for their roles as producers for expressing various recombinant proteins of importance. Thus, FP labeling systems have potentially broader applications than plasmid systems. Overall, this FP labeling system with the integration of *mcherry* is an important, non-invasive, and effective strategy for tracking and quantifying probiotics in various *in vivo* tissues as well as monitoring heterologous protein expression *in vitro*. Our results exemplify the research application potential of the integrated FP-labeled LAB strains as probiotics and bioengineering tools. Further investigation of the interaction between hosts and intestinal microbes using the FP labeling system and similar labeling techniques is underway.

## Data Availability Statement

The original contributions presented in the study are included in the article/supplementary material, further inquiries can be directed to the corresponding author/s.

## Ethics Statement

The animal study was reviewed and approved by the Animal Research Ethics Committee of Nanjing Normal University (Permit No. AREC 2018-10-26).

## Author Contributions

YY, WZ, WX, YC, HH, PW, and YL participated in the design and discussion of the study. WZ and YC carried out the experiments. WX and YC wrote the manuscript. YY and WX discussed, revised, and edited the manuscript. All authors read and approved the final version to be published.

## Conflict of Interest

WX was employed by company Geneception (Shanghai) Bio-technology Co., Ltd. The remaining authors declare that the research was conducted in the absence of any commercial or financial relationships that could be construed as a potential conflict of interest.
